# Population Code Dynamics in Categorical Perception

**DOI:** 10.1038/srep22536

**Published:** 2016-03-03

**Authors:** Chihiro I. Tajima, Satohiro Tajima, Kowa Koida, Hidehiko Komatsu, Kazuyuki Aihara, Hideyuki Suzuki

**Affiliations:** 1 Graduate School of Information Science and Technology, the University of Tokyo. 7-3-1 Hongo, Bunkyo, Tokyo 113-8656, Japan; 2 Department of Basic Neuroscience, University of Geneva. CMU, 1 rue Michel Servet, 1211 Genève, Switzerland; 3 EIIRIS, Toyohashi University of Technology. 1-1 Hibarigaoka, Tempaku, Toyohashi, Aichi, 441-8580, Japan; 4 National Institute for Physiological Sciences. 38 Nishigonaka Myodaiji, Okazaki, Aichi, 444-8585, Japan; 5 Institute of Industrial Science, the University of Tokyo. 4-6-1 Komaba, Meguro, Tokyo 153-8505, Japan

## Abstract

Categorical perception is a ubiquitous function in sensory information processing, and is reported to have important influences on the recognition of presented and/or memorized stimuli. However, such complex interactions among categorical perception and other aspects of sensory processing have not been explained well in a unified manner. Here, we propose a recurrent neural network model to process categorical information of stimuli, which approximately realizes a hierarchical Bayesian estimation on stimuli. The model accounts for a wide variety of neurophysiological and cognitive phenomena in a consistent framework. In particular, the reported complexity of categorical effects, including (i) task-dependent modulation of neural response, (ii) clustering of neural population representation, (iii) temporal evolution of perceptual color memory, and (iv) a non-uniform discrimination threshold, are explained as different aspects of a single model. Moreover, we directly examine key model behaviors in the monkey visual cortex by analyzing neural population dynamics during categorization and discrimination of color stimuli. We find that the categorical task causes temporally-evolving biases in the neuronal population representations toward the focal colors, which supports the proposed model. These results suggest that categorical perception can be achieved by recurrent neural dynamics that approximates optimal probabilistic inference in the changing environment.

We perceive sensory stimuli in two different ways: fine, or coarse as groups. For example, we can dissociate a group of red berries from the background green grass-field because of the rough color differences between them; at the same time, we can judge their maturities by discriminating between the slight differences in their colors. Recognizing very slight differences between stimuli, such as judging maturities of ripeness of fruits, is called *discrimination* (or *fine discrimination*). On the other hand, organizing stimuli within a certain region into one group, such as finding red berries in green grass, is called *categorization* (or *coarse discrimination*).

Classically, categorization has been considered as a “higher-order” cognitive process located at a later processing stage in contrast to other primitive sensory functions, such as discrimination of physical colors themselves. The neurons in higher areas express a more abstract level of information. For example, in color perception, color selective neurons in the ventral visual areas [including V1 and V4 and the inferior temporal (IT) cortex] show relatively smooth, continuous preference functions over the color space (called *hue*)^1^,^2^,^3^,^4^,^5^,^6^,^7^,^8^. These areas are also known to relate to fine color discrimination^1^,^2^,^4^. On the other hand, neurons in the frontal and parietal areas are reported to have discrete and categorical representations^9^,^10^. Such a hierarchical representation is consistently observed for a variety of stimulus features, including object shape and motion^11^,^12^,^13^,^14^,^15^,^16^,^17^. This hierarchical organization supports the view that categorical perception is achieved by sequential processes along the bottom-up information pathway from the sensory to higher areas.

On the other hand, recent empirical evidence suggests that the categorical cognitive structure can have profound effects on seemingly low-level perceptual processes. For example, linguistic knowledge, such as color names, has complex effects on color stimulus perception in human subjects^18^,^19^,^20^,^21^,^22^. Moreover, color perception has characteristic temporal dynamics. For example, recalled colors are gradually attracted toward the nearest categorical centers as time elapses after the stimulus offset^23^,^24^,^25^. These facts imply that the sensory percepts are shaped by dynamic and interactive mechanisms between the low-level sensory processes and the high-level, symbolic representations, rather than by a purely feedforward process from lower to higher stages. However, the detailed functions of the top-down signal from higher to lower stage are yet to be understood.

In the present study, we hypothesize that categorical stimulus information is processed based on a recurrent neural network, which extends the probabilistic population code^26^,^27^ with top-down and bottom-up connections among distinct neural populations. The probabilistic population code has been proposed as an effective scheme to represent probabilistic distribution of stimulus with neural ensemble activity^26^,^27^,^28^,^29^ although its biological plausibility in dynamic and high-dimensional inference problems is a recent topic of debate^30^. Here, we show that the probabilistic population code can approximate a dynamic hierarchical inference of stimulus and category when it is equipped with a recurrent interaction between neural populations at different levels. The model allows us qualitative and quantitative predictions on neural dynamics and perceptual performance. Remarkably, the model successfully accounts for a wide variety of physiological and cognitive phenomena as different aspects of a single system. Moreover, based on new electrophysiological data analyses, we confirm the key model dynamics in behaving monkey visual cortex.

## Results

### Categorical color perception as online statistical inference

We first describe our theoretical results by referring their relevance to categorical color perception. Note that the following results can be immediately generalized to categorical perception of any modality.

The first key idea of the present study is to describe categorical color perception in terms of online statistical inference. In natural environments, the chromaticity of a single point in a natural scene (during the execution of natural eye movements) tends to have characteristic dynamics, which consists relatively small fluctuations around several representative hues and discontinuous transitions among them. Suppose that the nervous system estimates hue value 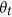 (e.g., a locus between red and green) according to the bottom-up sensory input 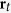 from the early sensory area at time 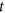. From the Bayes theorem, the posterior probability of a hue with a given sensory signal is given as follows:


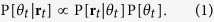


The first term in the right-hand side of Eq. (1) represents the likelihood of the sensory signal based on the stimulus hue, while the second term is the prior knowledge of the hue. To consider categorical effects, the prior probability of a hue is decomposed using a generative model in which the hue is stochastically generated from one of *n* color categories 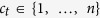 (e.g., “red” and “green”) as follows:


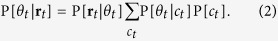


On the right-hand side, the second term is the probability of a hue based on the prior knowledge of its category, and the third term represents a further knowledge (referred to as *hyperprior*) of the category.

**Figure 1:**
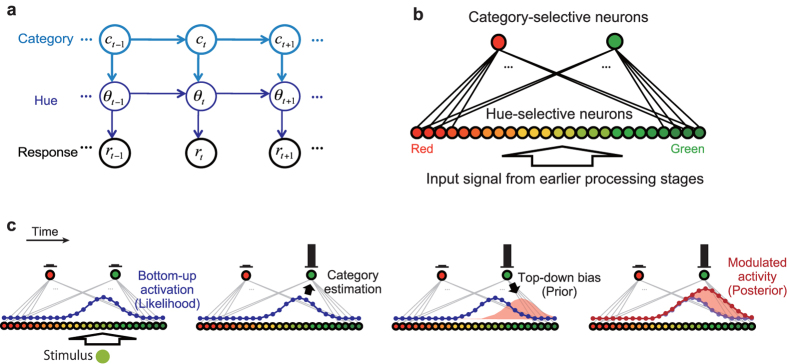
Model of online categorical inference. (**a**) Graphical model of chromatic stimulus observation. The neural response at each time is evoked by a hue value that is generated according to the color category. The arrows represent the probabilistic dependencies. (**b**) Architecture of the neural network that implements the statistical inference on the stimulus colors. The hue-selective neurons represent the continuous value of hue while the category-selective neurons represent the discrete color categories, such as “red” or “green.” The bottom-up input signal from the earlier stage is first received by the hue-selective neurons, and then the network decodes the hue and category through the interaction between two neural populations. We assume a one-dimensional hue space as a color space for simplicity, though the dimension can be extended to two or three. (**c**) Cycle of the modeled activity modulation in the network. The figure depicts four snapshots: from left to right, (i) the initial activity of the hue-selective neurons, (ii) the category estimation from the population activity of the hue-selective neurons, (iii) top-down bias signal based on the estimated category, and (iv) the modulated activity of the hue-selective population. The dotted curves above the hue-selective neuron layer represent the population activity, where the height of each dot schematically illustrates the magnitude of each neural activity. The activity magnitude of category-selective neurons are schematically indicated by the length of black bars above them.

We extend the above generative model to the time-varying category sequence, and derive an approximation for the online inference, which is the second key idea (Fig. 1a). In this case, the hue value 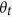 at time *t* is estimated from both present (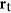) and past 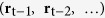 sensory signals. This can be achieved by an online inference algorithm for a hierarchical hidden Markov model^31^,^32^. A difficulty concerning the online inference is the calculation of the exact posteriors by the nested marginalization over the variables (see Supplementary Note A), which is computationally expensive and not likely to be implemented by the biological system *in situ*. However, we found that introducing a few reasonable approximations (e.g., the slowness of temporal variation in the category) yield a simplified representation of the log-posterior probability of hue, as follows:


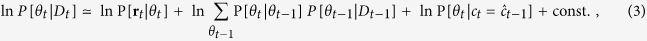


where 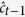 is the category estimated at time 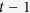, and 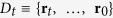 is the history of bottom-up signals. Here, the second and third terms represent prior knowledge that the hue value should be temporally continuous and that the presented hue should be similar to the expected categorical cluster, respectively (see Supplementary Note A). In the subsequent section, we refer to them as “continuity prior” and “categorical prior,” respectively.

### Recurrent neural network model for online categorical inference

The third key theoretical idea of this study is that we related the aforementioned mathematical formulation to activities of reciprocally connected neural populations. The calculation in Eq. (3) can be implemented by an extended probabilistic population code^26^,^27^, which is composed of two different groups of color-coding neurons: *hue-selective neurons* and *category-selective neurons* (Fig. 1b). Each hue-selective neuron receives an input signal from earlier processing stages, and has a narrow preference concentrated at a specific hue value, which is distributed homogeneously over the population. Each category-selective neuron has a wider preference that corresponds to a color category; the peak of the preference, a typical color of the category, is called the *category center* in this study. In this network model, the priors and the likelihood functions in Eq. (3) are represented by recurrent and sensory inputs into the hue-selective neural population. We modeled the top-down prior 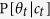 for category 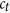 with von Mises distribution, and assumed that three categorical centers are distributed with equal spans across the whole hue circle. Note that we used the uniformly-spanned distribution of the categorical center for the sake of simplicity; it is straightforward to implement the non-uniformly located category centers such as reported in human color perception^33^,^34^. Assuming *Poisson*-like spike statistics, a population activity pattern can be linearly mapped to a log-likelihood with a specific stimulus value^26^,^35^,^36^,^37^,^38^, and the computation for a probabilistic cue combination is achieved by simple linear summations of the population activities^26^,^27^. Specifically, the iterative calculation of log-posterior in Eq. (3) is implemented by the following recurrence formula on the neural population activity:


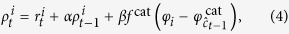


where 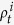 denotes the activity of the *i*th hue-selective neuron whose preferred color is 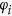; 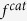 is a bell-shaped function peaked at 0; 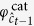 is the focal hue of the previously estimated category, 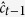; 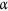 and 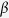 are parameters which reflect the certainty of prior expectations based on previous hue and category estimates. Three terms on the right-hand side represent (i) bottom-up input signals from the early visual stage, (ii) lateral self-feedback signals from the hue-selective neurons themselves, and (iii) top-down signals from the category-selective neurons, respectively. In the current model, the dependency of the hue-selective neurons’ activities on the previous ones of themselves also guarantees the continuous dynamics of neural activities across time steps; the continuous dynamics of neural population activity is also consistent with the neural dynamics in visual cortex as shown in a later section.

The modulation weights 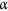 and 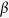 control the combining ratio, which reflects the predictabilities of the future hue stimuli based on the previous hue and category estimates, respectively. In our model 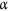 and 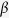 are effectively the only tunable parameters to replicate the results. The other model parameters, which reflect previous physiological data, do not affect the qualitative properties of the subsequent results. We used common parameter values throughout all the simulations (see also Supplementary Note A). Note that the primary goal of the present simulation is to replicate the qualitative aspects of a variety of phenomena, and the moderate changes in the model parameters do not affect the findings described in subsequent sections.

Figure 1c illustrates a single cycle of the iterative estimation process described by Eq. (4). The first step is to receive the bottom-up input signal at time *t* from the earlier visual stage (Fig. 1c, the first column). The hue-selective neurons receive inputs depending on their stimulus preferences: the cells preferring a hue similar to the presented stimulus receive large sensory inputs, 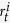. For example, with a greenish-yellow color stimulus, hue-selective neurons that have preferences nearer to the stimulus show the stronger responses, shaping a population activation pattern that is uniquely determined for each stimulus. The activities of the hue-selective neurons are propagated to the category-selective neurons through the bottom-up connection (Fig. 1c, the second column). Only the 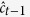
th category-selective neuron that received the strongest input at time 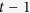 fires, according to a mutual competition, and generates a top-down signal to the hue-selective neurons (Fig. 1c, the third column). The function 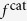 determines the connection weights between the category-selective neurons and the hue-selective neurons (see Supplementary Note A for the details). Finally, the updated population activity, which represents the posterior probability distribution on the hue value, provides a continuity prior in the next time step (Fig. 1c, the fourth column). These top-down and bottom-up processes estimate hue values and categories iteratively in the neural network. Note that this iteration is different from simple evidence accumulation for static noisy input, as was proposed previously^27^, in that the present study focuses on capturing more general time-varying stimulus dynamics.

### Response modulation, clustering, memory dynamics, and discriminability

The present model accounts for a wide variety of reported phenomena concerning categorical stimulus processing, based on a single mechanism. Here, we introduce the replication of four phenomena that were independently reported in previous studies: (i) task-dependent modulation of neural response, (ii) clustering of neural population representation, and (iii) temporal evolution of perceptual color memory, and (iv) a non-uniform discrimination threshold.

#### (i) Gain modulation of single-unit activities

**Figure 2:**
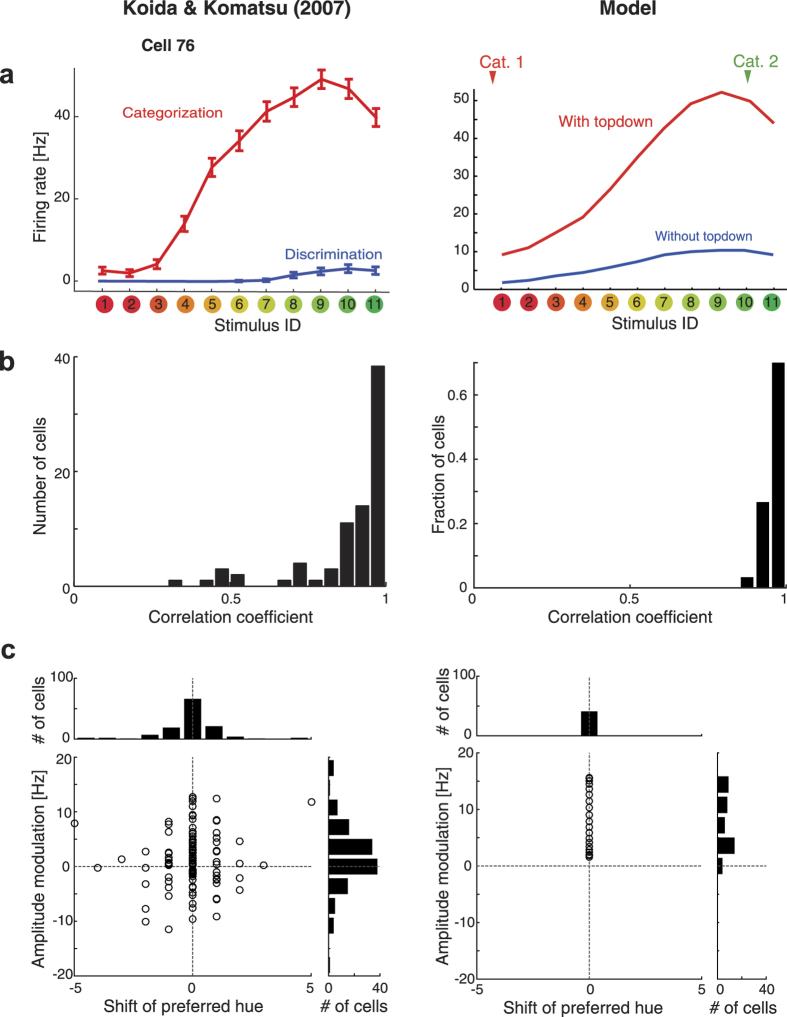
Model replicates the tuning modulations in color selective neurons in visual cortex. (Left) Tuning curve data from a macaque IT cortex. Data from Ref. 39. (Right) Effect of the top-down modulation on the tuning curve of a single neuron in the model. (**a**) Tuning curve of a single representative neuron. In the data, the response gain is modulated by the task demands, corresponding to the behavior of the model neuron. In the model, the top-down signal modulates the response gains of individual neurons. The error bars indicate the standard error of mean across trials (N = 16). (**b**) The stability of the stimulus selectivity in individual neurons. The plots show the distributions of Pearson’s correlation coefficient between the tuning curves obtained in categorization and in discrimination for each neuron. A coefficient 1 indicates that the neuron had identical stimulus selectivity during the categorization and discrimination tasks. (**c**) The scatter plots compare the modulation of response amplitude (mean response over all the stimuli) with the shift in preferred stimulus (the sample color that evoked the largest response in each cell). Each circle represents a single neuron. The histograms above the scatter plot show the distribution of the preferred stimulus shift; the histograms on the right show the distribution of the response amplitude modulation. In the experimental data (the left panels), effects were quantified by the differences between the categorization and discrimination tasks (categorization – discrimination). In the simulation (the right panels), the effect was quantified by the difference between with and without top-down effect (with top-down – without top-down).

First, the model provides a functional interpretation of task-dependent activity modulation in IT color-selective neurons at the single-unit level^39^. In a real neuron, the response amplitudes tended to be greater while the subjects performed color categorization, compared with when they discriminated fine differences in the presented colors. Notably, this task dependency has gain-modulation-like characteristics: the task demand does not affect the color preference, maintaining the relative tuning shapes almost invariant among conditions (Fig. 2a, left). In the model, the magnitude of the top-down signal has profound effects on single unit activities. We assume that the effect of the categorical prior, controlled by parameter 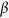 in Eq. (4), is relatively strong in the categorization task than in the discrimination task. This assumption is natural because the subject in the former task must focus more on categorical structures (where each stimulus hue belongs to one of categorical options) than on the fine differences in hue values. In the present problem setup with the hierarchical generative model, the categorical structure is represented in the prior probability distribution of hue, and is implemented by the top-down signal. The stronger top-down signal leads to larger neural activities (Fig. 2a, right), being consistent with the behavior of the real neurons.

What is non-trivial here is that the model also replicates the property similar to gain modulation as a net result of change in the recurrent connectivity. Categorical estimates that produce the top-down modulatory signals are determined depending on the activity of the hue-selective population itself, forming a positive feedback loop. It works as an approximately linear amplifier, and change in the feedback weight leads to a different amplification gain. As a result, the model predicts that the selectivity (shape of tuning curves except for gain) within each neuron was maintained almost identical across the task conditions even though the gain of response may depend on the task conditions (Fig. 2b, right). This is concordant with the property observed in the monkey visual cortex (Fig. 2b, left). We also confirmed directly that switching the task from discrimination to categorization led to the increase of neural response amplitude (*t*(124) = 3.4, P < 0.001) but no systematic shift in the preferred hue (*t*(124) = −0.59, P > 0.5; effect of variance explained by neurons’ preferred-hues: *F*(10,114) = 1.2, P > 0.3) (Fig. 2c, left). This was consistent with the model’s prediction (Fig. 2c, right). The relationship between the correlation coefficient and the gain modulation was not significant in our data (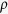 = −0.18, P > 0.05, Spearman rank correlation test; Supplementary Fig. S3).

#### (ii) Clustering effect

**Figure 3:**
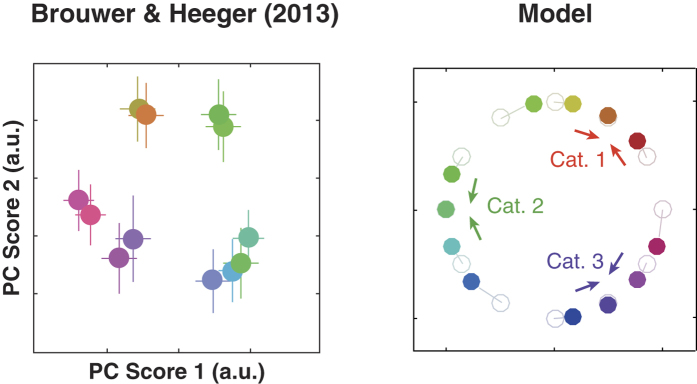
The model replicates clustering of population representation toward categorical centers under the categorical effects. (Left) Human VO1 responses to color stimuli, where multi-voxel patterns of fMRI neural activation were embedded in the first and the second principle components. Data replotted from Ref. 40. (Right) The model prediction. The vertical and horizontal axes represent a two-dimensional stimulus space, where the direction from the origin corresponds to stimulus hue while the deviation from the origin corresponds to the vividness of color (i.e., the origin corresponds to the white point). The colored dots indicate the stimulus properties represented by simulated neural population activities, by “decoding” the population activity [which is done by projecting the combinations of (peak locus, peak height) to the corresponding positions of this space]. Light-colored dots indicate the input stimuli while the dark-colored dots represent the neural population representation. The colors of markers correspond to those of presented stimuli. Here, we assumed three categories whose centers were in direction of 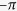, 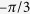, and 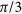 radians. The figure demonstrates that neuronal representations are biased towards the categorical centers. We used the same parameters as in Fig. 2 for consistency across the simulations; note that the strength of clustering depends on the magnitude of top-down interaction.

Second, the model replicates the structure of population activity in the visual cortex. Recently, Brouwer and Heeger^40^ measured cortical activity with functional magnetic resonance imaging (fMRI) while human subjects viewed different hues and performed a color-naming task. They reported that the neural color representation in human ventral visual areas, V4 and VO1 (but not V3), exhibited “clustering” (greater similarity between activity patterns evoked by stimulus colors within a perceptual category, compared to between-category colors) while the subject performed the color-naming task (Fig. 3, left). The clustering effect is naturally accounted for by the categorical modulation in the present model: in our model, the top-down modulation of activity is not uniform over the neural population, but is larger for units that prefer the hue near the category center. As a result, the patterns of neural population representation are more similar to each other for colors within the same category than for those straddling multiple categories (Fig. 3, right), being consistent with the clustering effect. In addition, our model explains a functional meaning of the clustering effect, as a consequence of optimal inference on the presented hue combined with prior knowledge that reflects the categorical structure of color.

#### (iii) Bias in memory color

**Figure 4:**
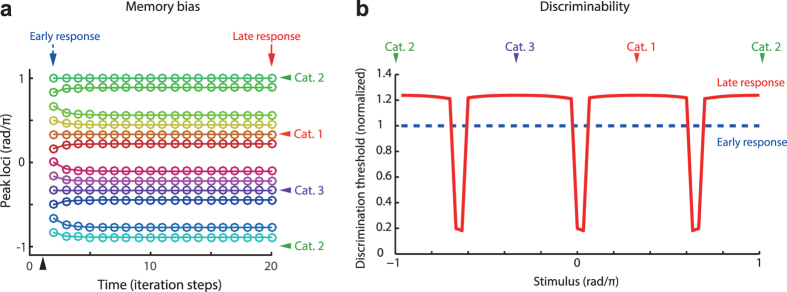
The model explains categorical effects on the bias and discrimination threshold in memorized color. (**a**) Temporal evolution of activity peak. The colored markers indicate the loci of peaks in the modeled neural population for each time step of simulation. The traces for 12 different stimuli are superimposed, where the colors of traces correspond to those of presented stimuli. The black arrow head on the horizontal axis indicates the timing of the stimulus onset. (**b**) The categorical effects on the stimulus discriminability. The solid curve indicates the threshold computed with the late response (time step = 20) after the stimulus onset; the dashed line indicates the threshold computed with the early response (time step = 2). The time points correspond to the solid and dashed arrows in panel a. The discrimination thresholds were normalized by that of the early response. Here the figures show the case in which three categorical centers were located uniformly on the hue circle. In panels a and b, the colored arrow heads with “Cat. x” indicate loci of the categorical centers.

Third, Fig. 4a shows the temporal evolution of hue values at the peak of population activity during a color memory task. Here, the preferred hue of the hue-selective neuron that showed the maximum magnitude at each time is plotted. Values at time 0 indicate the input hue presented at the initial point of the memory task; each hue-selective neuron is activated in response to the hue input at first. After time 0, hue-selective neurons received only the top-down signals from the category-selective neurons, and the input signals were set to zero. As the memory duration evolved, the represented hues approached the nearest category center. As it can be considered that the hue centers in the model correspond to the focal colors, these results agree with the characteristics of previously reported properties of memory color dynamics^23^,^24^,^25^. When the input hue is far from each category center, the model also reproduces the temporal increase in the difference between the memory color and the initially presented color^41^.

#### (iv) Categorical effect on discrimination performance

Fourth, the model also explained the nonuniform pattern of hue discrimination performance in human. The ideal observer analysis yields the discrimination threshold depending on the structure of hue category (Fig. 4b). The category dependency of discrimination performance is also understood intuitively as follows: the hue discriminability depends on the (signal) correlation of the activities of hue-selective neurons elicited by neighboring hue inputs. The two neural population responses respectively elicited by the two hues that lie in the same category are strongly correlated, whereas the correlation between those elicited by stimuli straddling two different categories is weaker. Therefore, the hue pairs belonging to the same category are more difficult to discriminate than those belonging to two different categories (Fig. 4b, solid curve). The results agree with the reported human psychophysical properties in delayed hue discrimination, where the subject discriminated sequentially presented hues^24^,^25^.

Notably, the categorical effect on the human hue discrimination is not always observed. For example, a recent study reports that no categorical effect was observed when the target and reference stimuli were presented simultaneously^42^, posing a question concerning whether and in which condition the category affects the human color discrimination. On the other hand, in the studies reporting the categorical effects^24^,^25^, the target and reference stimuli were often separated by a moderately long time interval (e.g., the reference stimuli were presented 2 s later from the two reference hues^24^). It required subjects to memorize the stimuli during each trial. In our model, no categorical effect was observed right after the stimulus onset (Fig. 4b, dashed line), being consistent with the study using simultaneous comparison^42^. In contrast, as mentioned above, the strong categorical effect was observed in the late period after the stimulus onset, due to the accumulating effect of the recursive update of the neural activity. These temporal dependency of categorical effect in the model reconciles the apparent contrast among the previous studies, suggesting that the categorical effect is a relatively slow process that is accounted for by the recurrent computation.

Note that we do not rule out the possibility that the discriminability of stimulus determined by an early visual system (e.g., retinal cones) affects the perceptual representation of categories, rather than vice versa. Instead, the present simulation demonstrates that, if we assume a neural circuit to implement categorical inference, such circuit mechanism affects the discriminability in a way that is consistent with human perceptual behavior. In particular, the latter framework (where category affects discriminability) provides a natural explanation for the human perceptual abilities in memory-guided stimulus discrimination, as described above.

### Dynamics of model neurons and macaque IT neurons

**Figure 5:**
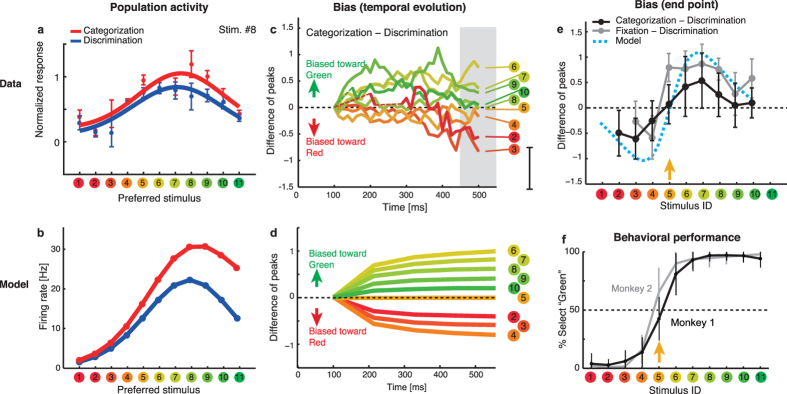
Comparing model behaviors with population dynamics in macaque IT cortex. (**a**) Modulation of the population activity during the categorization and discrimination tasks. The dots represent the average activity of neurons that prefer each of 11 stimulus colors. The curves are Gaussian fits of the population activity. The error bars indicate the standard errors of mean across neurons preferring each stimulus. (**b**) Activity distribution over the simulated hue-selective neural population (corresponding to panel a). The model predicts that the locus of maximum activity will shift toward the category center. (**c**) Dynamics of the peak loci in population activity, represented as differences between categorization and discrimination (the similar results were obtained for differences between fixation [passive viewing] and discrimination, Supplementary Fig. S2). The data are normalized by the difference at 100 ms after the stimulus presentation. The positive value indicates the shift toward green, and the negative value indicates the shift toward red. The units of the vertical axis is the difference in terms of the visual stimulus index (from stimuli 1 to 11). The color and number indicates the input stimulus hue. Stimuli #1 and #11 (two extremes in “red” and “green” directions) were omitted from the plot since the peak estimates with Gaussian fit were not reliable for those data due to the boundary effect. (**d**) Dynamics of peak hue modulations predicted by simulated neural population (corresponding to panel c). The error bar on the right of the plot indicates the bootstrap standard deviation (100 resampling) averaged over the stimuli and time bins. (**e**) Averaged peak loci for later responses (450–550 ms: the shaded area in panel c). The horizontal axis corresponds to the physical stimulus, while the vertical axis represents the difference in the peak loci for the categorization and discrimination tasks (the dark line), or in the fixation and discrimination tasks (see also Supplementary Fig. S1). The error bars are the bootstrap standard deviations (100 resampling). The blue curve depicts the peak hue shift predicted by the model (with category boundary located around stimulus 5, as indicated by the arrow). (**f**) Behavioral results in the categorization task. The arrow indicates the sample color corresponding to the categorical boundary. In these analyses, we analyzed the neural activities at or later than 100 ms after the stimulus presentation, because the earlier neural activities were weak and we could not obtain robust estimates for the peak loci of population activity.

To further investigate the relation between the model and an actual nervous system, we tested two key characteristics of the proposed model: (i) task-dependent modulation in the shape of entire population activity, and (ii) the dynamics of the modulation. The first characteristics is that the categorical top-down signal modulates the entire shape of the neuronal population response. To examine this hypothesis more directly, we analyzed the neural population activities recorded by Koida and Komatsu^39^. Although the authors reported that the individual single-cell activities of the color-selective neurons in the IT were modulated according to the task demands, it has been unclear how this modulation affected the stimulus representation of the entire cell population. To visualize the high-dimensional population response, we first classified neurons based on their preferred stimuli among 11 sample hues, and averaged the activities of neurons sharing the common stimuli after normalizing the activity by dividing them with the maximum response in the visual fixation task. For each time bin, this yielded neural population activity represented as an 11-dimentional vector, where each element corresponds to the average response of neurons preferring one of 11 sample stimuli. We fitted this averaged population activity by a Gaussian function over the preferred hues, and repeated this procedure for every time bin. Figure 5a shows the population response to a representative stimulus (stimulus No. 8, moderately greenish color). We found that the task demand modulated the neuronal activity at the population level. First, the amplitudes of the Gaussian fits for the categorization task were relatively higher than those for the discrimination task. Second, the peak of activity distribution tended to be biased toward the greenish extremes. Both properties were consistently observed among all sample stimulus colors (Supplementary Fig. S1). These results support the model predictions that the neuronal population representations were biased by the categorical top-down priors, resulting in the modulation toward focal colors (Fig. 5b). Note that the modulation did not change the stimulus selectivity in individual neurons as shown in Fig. 3. The bias is due to the heterogeneous gain modulation across the neural population, and it is observed only by reconstructing the entire population activity.

Next, we examined whether the model also accounts for the dynamics of the modulation in neural population activity. The analysis revealed that the dynamics of the categorical biasing effect depends on the presented stimulus hues (Fig. 5c,d). Although the polarity of bias is consistent with the model prediction, note that the population response peak in the neural data was not exactly located at the presented stimulus (Supplementary Fig. S1); therefore, in the subsequent analysis, we focus on the relative positions of the population response peaks between task-conditions. The differential analysis (Fig. 5e,f) suggested that the shift occurred toward either red or green, depending on the presented stimulus: the responses to reddish colors tended to be biased toward red, and the responses to greenish colors were biased toward green. This indicates that the polarity of the modulation was dynamically determined depending on the presented stimulus. Although the categorical effect in terms of population code is consistent with a previous report in object shape representation^17^, to the best of our knowledge, this is the first explicit demonstration of a peak hue shift in the population firing pattern that is flexibly modulated by the task-demand. Moreover, the shifts were found to gradually grow as time evolved after the stimulus presentation (Fig. 5c). The 3-way analysis of variance (ANOVA; 11 preferred stimuli × 11 presented stimuli × 2 time windows [early: 100–300 ms after the stimulus onset; late: 350–550 ms]) for trial-averaged activities in 125 cells indicated a significant effect of temporal evolution on the modulation strength (*F*(1, 2608) = 3.94, *P* < 0.05) and presented stimuli (*F*(10, 2608) = 16.29, *P* < 0.001). These results further support the idea that categorical modulations are related to the iterative process, as proposed in the present neural network (Fig. 1b). Notably, the categorical boundary suggested by this peak-shift analysis (Fig. 5e) was consistent with the behaviorally determined boundary of the category in the same experimental dataset (Fig. 5f), suggesting the functional relevance of the population activity modulation. Interestingly, we observed the same trends also for a fixation task, where the subject passively viewed the stimulus without explicit demand for categorization (Supplementary Fig. S2), suggesting that the dynamic categorical bias effect is a natural property of population dynamics of those color selective neurons. In both tasks, the peaks of population response shifted away from the category boundary (the categorization task, *t*(8) = 4.08, P < 0.005; the fixation task, *t*(7) = 4.08, P < 0.005; two-tailed t-test).

## Discussion

We proposed a hypothesis that categorical stimulus information is processed based on a recurrent neural network, which approximates an online hierarchical Bayesian estimation of stimuli. A previous study proposed a probabilistic population code that accounts for evidence accumulation in a network with unidirectional layer-to-layer interaction^27^. In the model, the high-level layer temporally sums up the activities of the lower layer via feedforward bottom-up connections, as was experimentally demonstrated in the parietal cortex^43^. Note that the present model is different from the previous model in that our model features the top-down interaction from high- to low-level layer that allows recursive and simultaneous update of category and stimulus estimates. That is, the reciprocal interaction between two different neural populations enables the present model to capture the stimulus that is generated from the dynamics of a hidden parameter (category). Remarkably, the present model of recurrent neural interaction was derived from a theoretical principle in the recursive Bayesian inference. The Bayesian argument clarifies the link between the normative inference and the neural network model, by demonstrating which aspect of the neural computation can correspond to each component in the optimal statistical inference; the sensory input, lateral interaction and top-down signal implement the likelihood, hue-based prior and category-based prior, respectively. Note that the details of network architecture could be arbitrarily designed without the optimality argument based on the inference theory (e.g., the theory of recursive Bayesian update constrained the architecture and functional forms of recurrent interactions between hue- and category-selective neurons, but we could consider the feedforward architecture with top-down interaction alone) although such network design is not always optimal for solving the inference problem. For example, a simple feedforward summation of the sensory input and the top-down signals (with some time-constants) are not optimal for inference based on a stimulus sequence, where the category and hue can change dynamically during the stimulus presentation (we will discuss later the predicted effect of stimulus dynamics on the neural representation and perception).

Although recurrent neural networks have been widely used in online recognition of category, such as speech recognition^44^,^45^, their relevance to the probabilistic representation by neural population code has not been emphasized in previous studies. A recent study models recursive update of stimulus estimate in Kalman filter by extending the probabilistic population code^46^, but the model does not consider the hierarchical dynamics of category and stimulus such as described in Fig 1a. In this study, using color category processing as an example problem, we demonstrated that the hierarchical Bayesian inference can be approximated by a relatively simple computation if we assume an extended architecture of the probabilistic population code. The Bayesian formulation was important because it enabled us not only to replicate the psychophysical phenomena but also to interpret their functions in a consistent framework. Moreover, implementation by population code allowed us quantitative comparison between model dynamics and recorded neural population data. It led us to the conclusion that a variety of cognitive biases concerning categorical perception are explained as a consequences of optimal statistical inference.

We implemented the recursive categorical inference by using the interactions between hue-selective and category-selective cell populations. The former is similar to what is typically found in the ventral visual areas, including V4 or IT^1^,^2^,^40^, while the latter is possibly represented in the prefrontal or parietal cortex^11^,^12^,^13^,^14^. An important point is that a relatively simple interaction among the different cell populations was found to be sufficient for implementing an approximated iterative Bayesian cue combination over a time sequence, where the presented color was estimated based on previous observations. Although, for simplicity, we assumed that the neural firing property in each population is fixed in the present model, it would be possible to extend the model to implement a more flexible representation of uncertainty by adding the effects of neuromodulators^31^,^32^.

The present model assumes that the strength of top-down interaction changes depending on task-context. The prefrontal cortex (PFC) is considered to be an important area for task switching, and possibly sends top-down, executive signals to other areas in a flexible manner depending on task-context^47^,^48^. Several mechanisms could implement the flexible modulation of top-down signals. First, the synaptic plasticity can be a source of the context-dependent modulation in top-down interaction, but we do not strongly believe that synaptic plasticity works as the major substrate of flexible and rapid behavior in the task-switching paradigm although short-term plasticity could account for task-dependency via the rapid changes in synaptic strength^49^,^50^, e.g., the monkeys could rapidly change their behavior depending on the task demand during the same session of recording. Second mechanism is the dynamic gating of neural signal transmission through dis-inhibitory gain control (e.g.,^51^). Dynamic inhibition of inhibitory interneurons at the target area of top-down connections may implement rapid gain control of the top-down signals. The third mechanism that potentially implements the rapid modulation of top-down signals is to flexibly suppress the activity of category-selective neurons depending on task-context. Note that, in the current model, inhibiting the category-selective neurons has the same effect as the gain modulation of top-down connection in terms of hue-selective neurons’ activities. These possibilities could be dissociated by future studies recording PFC neurons that represent stimulus categories.

We demonstrated that the model reproduced a wide variety of previously reported phenomena, which include the task-dependent single neuron activities, the clustering effect, the memory dynamics, and the category-dependent stimulus discriminability. These results suggest that the behavioral and physiological phenomena, which are separately reported in different research contexts, are interpreted as natural consequences of a unified model that approximates the statistically optimal computations concerning time-varying stimuli. The proposed theoretical framework could possibly be applied to similar phenomena found in other cortical areas involved in different sensory or feature modalities, such as sound^52^,^53^,^54^, visual motion^55^, object categorization^56^, or facial expression^57^.

Moreover, our model predicted that the dynamic modulation of neural population activity depended on all of the input stimuli, categorical top-down signals, and past estimation based on a hidden Markov model. We examined the plausibility of key model behaviors by analyzing activities of the IT neurons during categorization and discrimination tasks. The results of analysis of the neural activities recorded in the IT cortex were found to be consistent with the model behavior. These results provide the evidence of dynamic categorical effects on population coding in the cortex. It should be noted that the current analysis is based on sequential single-unit recording and does not capture the trial-to-trial noise-correlation among neurons. Although the structure of noise correlation can affect the quantitative aspect of stimulus discrimination performance, the currently known form of noise correlation in visual neurons is not likely to affect the qualitative results of the present analyses about categorical biases based on trial-averaged neural responses, such as shown in Fig. 5.

Interestingly, the categorical bias was observed not only in the categorization task but also in the fixation task (Supplementary Fig. S2). This suggests that the categorical prior is a default mode of sensory processing in IT cortex, at least in the color perception domain. In the fixation task, the subject only passively viewed the stimulus without any task; hence, theoretically, there is no explicit reason for subject to assume the categorical prior in this specific experimental task. However, in the natural environment, the categorical perception is important for detecting odd objects from a background based on coarse difference in visual features (e.g., a red venomous spider in a green grass field). In this sense, it is reasonable to use the categorical perception as a default mode of visual processing.

An alternative interpretation of the categorical bias in neural representation is that it is not the consequence of statistically optimal inference but simply reflecting the decision making by point-attractor dynamics^17^. In the latter view, the temporal evolution of neural population representation does not necessarily reflect the recursively-updated estimate on sensory stimulus; for example, it can be transient dynamics attracted to fixed points in associative memory, such as in Ref. 58. In this framework, the similarity of the categorization and passive fixation tasks and their distinction from the discrimination task have to be explained not by the categorical prior effect but by assuming some special processing required for fine discriminations in the discrimination task, although theoretical interpretation of such a special processing is not clear. To test whether the neural population activity reflects the recursive update of stimulus or transient dynamics with fixed-point attractors, we can introduce the temporal structure of stimulus during each trial. In particular, the stimulus change in a late period of visual stimulus presentation is expected to have a great effect on the recursive update of sensory information, but not on the transient dynamics.

When stimulus dynamically changes within each trial, the present recursive algorithm also makes a prediction that is somewhat counterintuitive: if the stimulus hue jumps from green to red, the responses of neurons selective to intermediate hues (neither red nor green) will transiently increase. The iterative update of hue-selective neuron activities causes a gradual shift in the hill of activity of the hue selective population (e.g., Fig. 1c) and the firing rate of the yellow-selective neurons increases. This offers a very strong, testable experimental prediction. This can be tested by future study that extends the current experiment by introducing temporal structure of stimulus.

Although the present model and experimental results suggest slow and continuous modulations of neural population activity, the earliest effect begins immediately after the stimulus onset (e.g., Figs 4a and 5c). These early categorical effects, possibly reflecting the categorical estimate based on the initial bottom-up signal, are relevant to the rapid perceptual categorization^59^,^60^ or category selectivity in visual neurons^61^, and also consistent with our previous report based on the single-neuron level analysis^39^. Note that, in the present model, the category was estimated at *every time step*, meaning that the category could be estimated not only after the neural activities at the attractor but also during the transient dynamics. Therefore, the recurrent computation does not necessarily contradict with the rapid skills of perceptual categorization.

Beyond the domain of hue category, we could consider a situation such that a high-level category is defined by a combination of distinct multiple clusters of stimuli, which is interpreted as a ‘category of category’. Such a complex structure in category leads to multimodal prior function 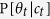 of hue 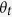. In this case, the simple computation described by Eq. (4) does not always approximate the optimal inference. To deal with such a complex categorical structure, we can consider an extended network model based on a similar idea but with a deeper hierarchy that represents ‘category of category’. Inference with deeper categorical structure is an interesting direction of future extension of the model.

The most important characteristic of the proposed information-processing scheme is dynamic estimation with top-down categorical operation. Although there have been previous attempts to relate categorical effects and a Bayesian prior^62^, few studies have focused on the dynamic aspect of categorical computation. The present model shares the idea with the studies that propose the contribution of top-down interaction that modifies the sensory representation and affects the choice probability^63^,^64^,^65^. These studies that put emphasis on bidirectional interaction among cortical areas via top-down and bottom-up interactions challenge the classical view of sensory processing where the information flows directionally from the low- to high-level modules. From the viewpoint of online information processing, we propose that the recurrent hierarchical computation with stimulus category benefits the brain at least with three advantages: (i) information compression, (ii) accurate and stable estimation, and (iii) short-term information retention. First, the discretized category can be utilized as a compressed representation of the stimulus. A high capacity is required to record all responses of the hue neurons to input signals (i.e., recording with the resolution of the repertoire of the hue-selective units), and it is not efficient to share such representations across brain areas or to memorize them for a long time. In addition, it is also not effective to retain for a long time the precise values of stimuli when the input signals are noisy and fluctuate rapidly. The compressed representation with category is considered to save a large amount of the storage capacity in the brain in exchange for a small amount of information loss. Second, the sequential Bayesian framework based on the past estimation enables an accurate and stable estimation. The estimation by combining a top-down, bottom-up, and recurrent components allows the system to track the stimulus under a changing environment. It is also straightforward to adapt to different environments by altering the weights on those components according to the uncertainty of each signal. Third, the recurrent interaction naturally implements the short-term memory of stimulus. Since the two neural populations in our model interact with each other through excitatory connections, the positive feedback maintains the neural activity for a short duration with a decay constant after the stimulus offset. These three advantages imply a possibility that a single system can serve as a basis of conceptually different functions. The present model and analysis demonstrate that, at least in categorical processing, the apparently divergent cognitive and physiological phenomena can be explained as consequences of a single computation within a network.

## Methods

### Simulation of neural network model

We implemented the estimation algorithm in the previous section by a neural network model with 300 hue-selective neurons and 3 category-selective neurons. For simplicity in this study, we assumed a one-dimensional hue space as a color space, though the dimension can be extended to two or three. We employed the von Mises function, 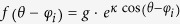, with a sharpness parameter 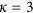 and gain 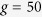, for the tuning curve 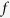 of the hue-selective neurons; these parameters roughly replicates the color selectivity in IT neurons^39^. Although we used the von Mises function as tuning curves for the consistency with the theoretical analysis described in the previous sections, we could use an arbitrary bell-shaped function (for example, similar discussion on the approximated optimality applies to Gaussian, where we assume quadratic functions instead of sinusoidal functions for the likelihood computation; see Supplementary Note A). In the simulation, we assumed 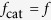 for simplicity. We also implemented the noise in the input signal to the hue-selective neurons with a Gaussian having standard deviation of 10 azimuth degree in the hue circle, which is roughly matched to the previously reported hue-discrimination threshold (for ~79% correct discrimination of color patches) in human subjects^42^ (changing the amount of noise did not affect the qualitative predictions by the model).

#### Bottom-up signal

The category 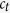
was estimated by approximately maximizing the log-likelihood of the population activity of the hue neurons. The maximum likelihood estimate was provided via a winner-take-all competition among the category-selective neurons, where the category-selective neurons’ initial activities were given by the linear weighted sums of the activities of the hue-selective neurons as follows:


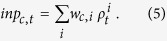


Here, 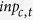 represents the inputs to the *c*th category-selective neuron, and the weight is given as 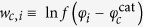. Note that the activities of category-selective neurons were computed based on the bottom-up signals from hue-selective neurons, which had relatively slow dynamics due to their dependency on the previous states of themselves via the lateral connections. This made the category estimate stable even though the winner-take all competition was repeated in every time step.

#### Top-down signal

The hue presented at time *t* was estimated based on the knowledge of hue at time *t*−1. Given the assumption that the hue presented in the future was likely to be generated from the same category as the present one, the posterior probability was expressed with the Bayes formula in Eq. (1). The information of the prior probability distribution 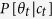 was given by a top-down signal from a category-selective neuron to the *i*th hue-selective neuron: 
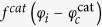
.

#### Ideal observer analysis

The hue discrimination threshold with the modeled network was derived based on the standard ideal observer analysis^37^,^66^,^67^,^68^. The Fisher information of population activity around a hue-value 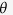
is defined by 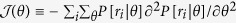. In our model, it is simplified as 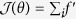 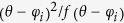. With the ideal stimulus decoder, the asymptotic discrimination threshold, in a large population size limit, is given by 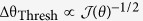.

### Electrophysiological recording and data analysis

All procedures for animal care and experimentation were in accordance with the National Institutes of Health Guide for the Care and Use of Laboratory Animals and were approved by Institutional Animal Experimentation Committee. Details of surgical and recording procedures have been previously published^39^ and also described in Supplementary Note B. Two monkeys (*Macaca fuscata*) were used for the experiments. The monkeys were trained in a categorization task, a discrimination task and a simple fixation task. In all three tasks, 11 sample colors were presented in a pseudorandom order. The monkey was required to maintain fixation within the trial, except for the saccade response. The sample color stimulus was presented for 500 ms. There were eleven sample colors that ranged from red [color 1, (x, y) = (0.631, 0.343)] to green [color 11, (x, y) = (0.286, 0.603)] with equal spaces on the CIE xy chromaticity diagram. We recorded and analyzed 125 single neurons in area TE (the anterior part of IT cortex).

#### Categorization task

The monkey reported whether the sample color was reddish (sample colors 1–4) or greenish (sample colors 8–11) by saccade, and was rewarded for correct responses. For the intermediate colors (sample colors 5–7), the monkey was rewarded randomly regardless of its behavioral response.

#### Discrimination task

The monkey reported which of test color was the same as the reference color by saccade. The two choice colors were three steps apart along the 11 sample colors: the eight choice color pairs included colors 1–4, 2–5, 3–6, 4–7, 5–8, 6–9, 7–10 and 8–11. This color interval was chosen so as to yield a modest performance (about 80–90% correct).

#### Recording

Neuronal activity was recorded from the anterior part of the IT cortex, which is a region where color-selective neurons are concentrated. To record single unit activities, microelectrodes were inserted, and the activities of single neurons were isolated by matching spike templates.

## Additional Information

**How to cite this article**: Tajima, C. I. *et al*. Population Code Dynamics in Categorical Perception. *Sci. Rep*. **6**, 22536; doi: 10.1038/srep22536 (2016).

## Supplementary Material

Supplementary Information
