# Supplementary material for: Population Code Dynamics in Categorical Perception
Source: Sci Rep. 2016 Mar 3;6:22536. doi: 10.1038/srep22536 (PMC4776180; doi:10.1038/srep22536)
Supplement: Supplementary Information [file srep22536-s1.pdf]

Population Code Dynamics in Categorical Perception

Chihiro I. Tajima[a,*], Satohiro Tajima[b,*], Kowa Koida[c], Hidehiko Komatsu[d], Kazuyuki Aihara[e,a], and Hideyuki Suzuki[a]

a. Graduate School of Information Science and Technology, the University of Tokyo. 7-3-1 Hongo, Bunkyo, Tokyo 113-8656, Japan.
b. Department of Basic Neuroscience, University of Geneva. CMU, 1 rue Michel Servet, 1211 Genève, Switzerland.
c. EIIRIS, Toyohashi University of Technology. 1-1 Hibarigaoka, Tempaku, Toyohashi, Aichi, 441-8580, Japan.
d. National Institute for Physiological Sciences. 38 Nishigonaka Myodaiji, Okazaki, Aichi, 444-8585, Japan.
e. Institute of Industrial Science, the University of Tokyo. 4-6-1 Komaba, Meguro, Tokyo 153-8505, Japan.

*These authors contributed equally to this work.

Corresponding authors: CIT: chihi.at.heart@gmail.com; ST: satohiro.tajima@gmail.com, Department of Neuroscience, University of Geneva. CMU, 1 rue Michel Servet, 1211 Genève, Switzerland.

Supplementary Information

Contents

Supplementary Note 2
A. Neural network model that approximates dynamical categorical inference 2
Step1: Exact statistical inference 2
Step 2: Approximated statistical inference 3
Step 3: Implementation by recursive population code 4
B. Electrophysiological recording and data analysis 11
References 13
Supplementary Figures 14
Supplementary Figure S1. 14
Supplementary Figure S2. 15
Supplementary Figure S3. 16

Supplementary Note

A. Neural network model that approximates dynamical categorical inference

We formulate the categorical perception as an online inference problem, and we propose a neural network model that works as an inference algorithm approximately. While the problem formulation and the model themselves are presented in **Results** in main text, we explain in this Supplementary Note the reason why the model works as a hierarchical Bayesian inference algorithm; namely, we provide technical details as to (i) how the exact inference algorithm is derived from the problem formulation, (ii) how the approximated algorithm is derived, and (iii) how the approximated algorithm is implemented in a biologically plausible neural network.

We begin by deriving the optimal statistical inference of the stimulus category, where the category and the sensory evidence may change across time. We consider the task that a higher visual area infers stimulus value θ_t and its category c_t at time *t* from the history of bottom-up signals $D_t \equiv \{\mathbf{r}_t, \ldots, \mathbf{r}_0\}$ transmitted from the early visual system until time *t*, where there are a given number (n) of categories $c_t \in \{1, \cdots, n\}$. To solve this problem, we assume that the response activities $\mathbf{r}_t$ of sensory neurons are received from the early visual system. The temporal evolution of the stimulus value θ_t is dependent on its category c_t and follows the graphical model shown in **Fig 1a**.

Step1: Exact statistical inference

The posterior probability of stimulus (e.g., hue of color) θ_t is expressed as follows:

$$\mathrm{P}[\theta_t|D_t] = \mathrm{P}[\theta_t|\mathbf{r}_\mathrm{t}, D_{t-1}] \propto \mathrm{P}[\mathbf{r}_t|\theta_t]\ \mathrm{P}[\theta_t|D_{t-1}]. \tag{S1}$$

Considering the marginalization and dependencies among the variables, we have

$$\mathrm{P}[\theta_t|D_t] \propto \mathrm{P}[\mathbf{r}_\mathrm{t}|\theta_t] \sum_{c_t} \sum_{\theta_{t-1}} \mathrm{P}[\theta_t|\theta_{t-1}, c_t]\ \mathrm{P}[\theta_{t-1}|D_{t-1}] \sum_{c_{t-1}} \mathrm{P}[c_t|c_{t-1}]\ \mathrm{P}[c_{t-1}|D_{t-1}]. \tag{S2}$$

This representation of the posterior contains deeply nested summations. Given the potential computational cost and restrictions on the nervous system, it is not likely that the biological system implement the calculation shown in Eq. (S2) *in situ* [1]. In the subsequent sections, we derive an approximated representation of the posterior.

Step 2: Approximated statistical inference

We propose a reduced estimation scheme that approximates the exact inference shown above. We assume that a neural population receive sensory input signals containing stimulus information and noise. The purpose of the model is to estimate the true stimulus identity without noise. We introduce the following three approximations:

(a1) *The category slowly changes compared to the stimulus*:

$$\mathrm{P}[c_t|c_{t-1}] \simeq \delta_{c_t,c_{t-1}}. \qquad \text{(S3)}$$

In natural environment, it is reasonable to assume that the stimulus can change, but the category rarely changes over time except for drastic changes as explained above. For example, appearance of object surface can be determined by the surface reflectance property and the lighting condition. Although the lighting condition (e.g., the surface angle) can alter in a short time, the surface property (e.g., reflectance spectra) is often relatively constant. Therefore, it is relatively rare to observe a drastic appearance change straddling a categorical boundary (e.g., such that a red object suddenly becomes blue) even though the precise appearance of surface may change rapidly.

(a2) *Omitting the uncertainty of the previous category estimate*:

$$\mathrm{P}[c_{t-1}|D_{t-1}] \simeq \delta_{c_{t-1},\hat{c}_{t-1}}, \qquad \text{(S4)}$$

where $\hat{c}_{t-1}$ is the categorical estimate at time $t-1$. As described later, in the simulation, we provided the categorical estimate at each time point by maximizing the likelihood of sensory input.

(a3) *Variable separation*: The posterior probabilities of an estimated stimulus, conditioned by θ_{t-1} and c_t, were assumed to be approximated as

$$\mathrm{P}[\theta_t|\theta_{t-1}, c_t] \propto \mathrm{P}[\theta_t|\theta_{t-1}]\ \mathrm{P}[\theta_t|c_t]. \qquad \text{(S5)}$$

This approximation is valid in many natural situations, for example, where $\mathrm{P}[\theta_t|\theta_{t-1}, c_t]$, $\mathrm{P}[\theta_t|\theta_{t-1}]$, and $\mathrm{P}[\theta_t|c_t]$ are all Gaussian-like unimodal distributions and $\mathrm{P}[\theta_t|\theta_{t-1}, c_t]$ is maximized at a point between the peaks of $\mathrm{P}[\theta_t|\theta_{t-1}]$ and $\mathrm{P}[\theta_t|c_t]$.

Although these three assumptions are natural for realistic environments, it should be noted that they do not always hold,

especially in laboratory experiments. For example, in an experiment where the experimenter can use a completely unstructured stimulus sequence, the slower dynamics of category compared to that of hue, Eq. (S3), could deviate from the reality. In this sense, the inference based on this assumption becomes sub-optimal in such an artificial situation. Nevertheless, it is possible that the brain, which could be adapted to the natural environment, still uses this sub-optimal inference strategy during the laboratory experiment. Similarly, the assumption described by Eq. (S5) is not always valid, for example, when $\mathrm{P}[\theta_t|\theta_{t-1}, c_t]$ is not maximized between the peaks of $\mathrm{P}[\theta_t|\theta_{t-1}]$ and $\mathrm{P}[\theta_t|c_t]$. This could occur when the experimenter artificially introduces a complex structure of category such that $\mathrm{P}[\theta_t|c_t]$ is a multimodal function of hue θ_t. The inference under such the complex categorical structure is beyond the scope of the present study although it is an interesting direction of future extension of the current model.

Applying the above approximations (a1)–(a3) to Eq. (S2), we have:

$$\mathrm{P}[\theta_t|D_t] \propto \mathrm{P}[\mathbf{r}_t|\theta_t]\ \textstyle\sum_{\theta_{t-1}} \mathrm{P}[\theta_t|\theta_{t-1}]\, \mathrm{P}[\theta_{t-1}|D_{t-1}]\, \mathrm{P}[\theta_t|c_t = \hat{c}_{t-1}], \tag{S6}$$

or, equivalently,

$$\ln \mathrm{P}[\theta_t|D_t] \simeq \ln \mathrm{P}[\mathbf{r}_t|\theta_t] + \ln \sum_{\theta_{t-1}} \mathrm{P}[\theta_t|\theta_{t-1}]\, \mathrm{P}[\theta_{t-1}|D_{t-1}] + \ln \mathrm{P}[\theta_t|c_t = \hat{c}_{t-1}] + \mathrm{const.} \tag{S7}$$

This corresponds to Eq. (3) in **Results** in main text.

Step 3: Implementation by recursive population code

Next, we explain how the above online statistical inference can be achieved with a recurrent neural network (the parameters used in the simulation are summarized in **Table S1**). Let r_t^i denote each bottom-up, stimulus-evoked spike signal (hereafter, we refer to it as *sensory input*), received by the ith hue-selective neuron at time point t. We assume that the sensory inputs to individual neurons, $\mathbf{r}_t = (r_t^1, \dots, r_t^N)$, are generated by an independent Poisson process for a given stimulus value, θ_t as follows:

$$\mathrm{P}[\mathbf{r}_t|\theta_t] = \prod_i \frac{f(\theta_t - \varphi_i)^{r_t^i}}{r_t^i!} e^{-f(\theta_t - \varphi_i)}, \tag{S8}$$

where $f(\theta_t - \varphi_i)$ is the expected spike count between steps $t-1$ and t in the ith hue-selective neuron, and φ_i denotes its preferred stimulus. (Here we assume the independence of spike-count variability in $\mathbf{r}_t$ given the input to each neuron, but this should be discriminated from the apparent noise correlation that may include fluctuations in shared network input to each neuron. The assumption of independent Poisson process does not rule out such the correlated variability reflecting the correlation in network input to neurons; rather, it focuses on the variability within each neuron after eliminating the global fluctuation that

originates from shared signals [2].)

If we assume that the preferred stimulus is uniformly distributed across the neural population, the log-likelihood of the given sensory input is simplified as:

$$\ln \mathrm{P}[\mathbf{r}_t|\theta_t] = \sum_i r_t^i \ln f(\theta_t - \varphi_i) + \text{const.} \tag{S9}$$

Here, the constant term is independent of θ_t. We used the fact that the summation $\sum_i f(\theta_t - \varphi_i)$ does not depend on θ_t due to the uniformity when the total number of neurons is sufficiently large. (Note that the concept of uniformity depends on the choice of the stimulus space; we assume a stimulus space that approximately satisfies this condition. In addition, the uniformity assumption about neuronal selectivity often does not hold for intensity dimensions, such as stimulus contrast.) Notably, Eq. (S9) implies a mapping relationship from a population input $\mathbf{r}$ to the logarithm of the distribution function of stimulus θ, through the linear summation of kernels, $\ln f(\theta_t - \varphi_i)$ [3–7]. Assuming a generic nonlinear function f and a sufficient number of neurons that prefer different stimulus values, arbitrary functions of θ can be mapped to the space of the population input $\mathbf{r}$. Specific constraints on f lead to the restriction on the image of this map, but the image includes a family of functions practically sufficient to encode posterior distributions of the stimulus under the biologically plausible assumptions explained below.

To be specific, we introduce parametrized models of the neural tuning function and the probability distributions. First, we assume that the neural tuning function *f* is a von Mises function (circular Gaussian), $f(x) = \exp(\kappa \cos x)$, with a tuning sharpness parameter κ. Second, we consider the situation in which the stimulus transition is described as $\mathrm{P}[\theta_t|\theta_{t-1}] \propto \exp(\sigma \cos(\theta_t - \theta_{t-1}))$ with a relatively large sharpness σ. Third, the top-down prior $\mathrm{P}[\theta_t|c_t = \hat{c}_{t-1}]$ is described by a von Mises distribution with an arbitrary sharpness κ^{cat} and a mode (or *focal stimulus*) $\varphi^{\mathrm{cat}}_{\hat{c}_{t-1}}$ that corresponds to the previously estimated category $\hat{c}_{t-1}$:

$$\mathrm{P}[\theta_t|c_t = \hat{c}_{t-1}] \propto \exp\left(\kappa^{\mathrm{cat}} \cos\left(\theta_t - \varphi^{\mathrm{cat}}_{\hat{c}_{t-1}}\right)\right). \tag{S10}$$

At the very beginning of inference, it is reasonable to assume that the nervous system has no prior information about the stimulus, and that the activities of the hue-selective neurons $\boldsymbol{\rho}_t = (\rho_t^1, \ldots, \rho_t^N)$ at time t represent the likelihood function simply reflecting the sensory inputs: $\rho_t^i = r_t^i \ (\forall i)$. Our basic idea in the present study is that we can also represent the posterior

distribution of θ by modifying the population input $\mathbf{r}$ in Eq. (S9) to reflect prior distributions that depend on previous input history and on top-down signals, corresponding to the second and the third terms in Eq. (S6); that is, there exist vectors $\boldsymbol{a}_t = (a_t^1, \dots, a_t^N)$ and $\boldsymbol{b}_t = (b_t^1, \dots, b_t^N)$ that satisfy

$$\ln \sum_{\theta_{t-1}} \mathrm{P}[\theta_t|\theta_{t-1}]\, \mathrm{P}[\theta_{t-1}|D_{t-1}] = \sum_i a_t^i \ln f(\theta_t - \varphi_i) + \text{const.}, \tag{S11}$$

$$\ln \mathrm{P}[\theta_t|c_t = \hat{c}_{t-1}] = \sum_i b_t^i \ln f(\theta_t - \varphi_i) + \text{const.} \tag{S12}$$

Then, Eq. (S7) is rewritten as follows:

$$\ln \mathrm{P}[\theta_t|D_t] = \textstyle\sum_i \rho_t^i \,\ln f(\theta_t - \varphi_i) + \text{const.}, \tag{S13}$$

$$\rho_t^i = r_t^i + a_t^i + b_t^i, \tag{S14}$$

where ρ_t^i is the updated activity of the ith hue-selective neuron at time t. In this equation, the product of the distribution functions $\mathrm{P}[\mathbf{r}_t|\theta_t]\, \mathrm{P}[\theta_t|c_t = \hat{c}_{t-1}] \sum_{\theta_{t-1}} \mathrm{P}[\theta_t|\theta_{t-1}]\, \mathrm{P}[\theta_{t-1}|D_{t-1}]$ is substituted by the linear sum, $r_t^i + a_t^i + b_t^i$, where a_t^i and b_t^i are interpreted as bias inputs to the neuron i. Now, the problem is to derive the appropriate functional forms of a_t^i and b_t^i.

First, we show that, b_t^i is expressed in the following form:

$$b_t^i = \beta f^{\text{cat}}\left(\varphi_i - \varphi_{\hat{c}_{t-1}}^{\text{cat}}\right), \tag{S15}$$

with an arbitrary even function f^{cat}if β satisfies the following condition:

$$\beta = \frac{\kappa^{\text{cat}} \cos\left(\theta_t - \varphi_{\hat{c}_{t-1}}^{\text{cat}}\right)}{\kappa \sum_i f^{\text{cat}}\left(\varphi_i - \varphi_{\hat{c}_{t-1}}^{\text{cat}}\right) \cos(\theta_t - \varphi_i)} = \frac{\kappa^{\text{cat}}}{\kappa \tilde{f}^{\text{cat}}}, \tag{S16}$$

where we defined $\tilde{f}^{\text{cat}}$ as the cosine coefficient in the Fourier transforms of f^{cat}. To see this, using Eqs. (S10) and (S15) and the definition of tuning function, $f(x) = \exp(\kappa \cos x)$, (S15) can be rewritten as

$$\kappa^{\text{cat}} \cos\left(\theta_t - \varphi_{\hat{c}_{t-1}}^{\text{cat}}\right) = \sum_i \beta f^{\text{cat}}\left(\varphi_i - \varphi_{\hat{c}_{t-1}}^{\text{cat}}\right) \kappa\cos(\theta_t - \varphi_i), \tag{S17}$$

ignoring the constant term that is irrelevant to the hue estimation. This gives the first.equality in Eq. (S16). To derive the second equality in Eq. (S16), we can rewrite the summation in denominator of the middle term as $\sum_i f^{\text{cat}}\left(\theta - \varphi_{\hat{c}_{t-1}}^{\text{cat}} - \psi\right) \cos\psi$ by

defining $\psi = \theta - \varphi_i$. From the uniformity of preferred-stimulus distribution across neurons, this summation is interpreted as the convolution between f^{cat} and $\cos$, and thus simply expressed as $\tilde{f}^{\mathrm{cat}} \cos\left(\theta - \varphi^{\mathrm{cat}}_{\hat{c}_{t-1}}\right)$ with the cosine coefficient $\tilde{f}^{\mathrm{cat}}$, using that f^{cat} is an even function. Now, the θ-dependent term, $\cos\left(\theta - \varphi^{\mathrm{cat}}_{\hat{c}_{t-1}}\right)$, cancels out between the numerator and the denominator, providing the second equality in Eg. (S16). Therefore, we have a θ-independent expression, $\beta = \kappa^{\mathrm{cat}}/\kappa\tilde{f}^{\mathrm{cat}}$, and there exist β that satisfies this condition. Equation (S16) illustrates that the gain of the bias input should be determined as proportional to the certainty of the prior; e.g., when the top-down prior knowledge is certain, the sharpness parameter κ^{cat} of the distribution function $\mathrm{P}[\theta_t|\hat{c}_{t-1}]$ has a large value, and also the gain of top-down bias β should be large. In the simulation, we assumed $\kappa^{\mathrm{cat}} = \kappa$ for the simplicity although κ^{cat} is not necessarily equal to κ if the Eq. (S16) is satisfied.

Next, we derive a_t^i. Similarly to Eq. (S9), the log-posterior of the past stimulus is expressed (using the von Mises tuning property and the neural activity ρ_{t-1}^i) as

$$\ln \mathrm{P}[\theta_{t-1}|D_{t-1}] = \sum_i \rho_{t-1}^i \cos(\theta_{t-1} - \varphi_i) + \mathrm{const.} = \tilde{\rho}_{t-1} \cos\left(\theta_{t-1} - \hat{\theta}_{t-1}\right) + \mathrm{const.}, \tag{S18}$$

with $\hat{\theta}_{t-1}$, the mode of the distribution, and $\tilde{\rho}_{t-1}$, the cosine coefficient in the Fourier transforms of activity as a function of preferred stimulus: $\rho_{t-1}(\varphi_i) = \rho_{t-1}^i$. Eq. (S18) and $\mathrm{P}[\theta_t|\theta_{t-1}] \propto \exp(\sigma\cos(\theta_t - \theta_{t-1}))$ yield $\sum_{\theta_{t-1}} \mathrm{P}[\theta_t|\theta_{t-1}]\, \mathrm{P}[\theta_{t-1}|D_{t-1}] \propto \sum_{\theta_{t-1}} \exp(\sigma\cos(\theta_t - \theta_{t-1})) \exp\left(\tilde{\rho}_{t-1}\cos\left(\theta_{t-1} - \hat{\theta}_{t-1}\right)\right)$, where the convolution between two von Mises functions in the left-hand side is approximated, for sufficiently large σ and $\tilde{\rho}_{t-1}$, as

$$\begin{aligned} \ln \sum_{\theta_{t-1}} \mathrm{P}[\theta_t|\theta_{t-1}]\, \mathrm{P}[\theta_{t-1}|D_{t-1}] &\approx \frac{\sigma\tilde{\rho}_{t-1}}{\tilde{\rho}_{t-1} + \sigma} \cos\left(\theta_t - \hat{\theta}_{t-1}\right) + \mathrm{const.} \\ &= \frac{\sigma}{\tilde{\rho}_{t-1} + \sigma} \sum_i \rho_{t-1}^i \ln f(\theta_t - \varphi_i) + \mathrm{const.} \end{aligned} \tag{S19}$$

(To show the approximated equality, for example, we can consider a Gaussian approximation of von Mises function, such as $\exp(\sigma\cos(\theta_t - \theta_{t-1})) \propto \exp(-\sigma(\theta_t - \theta_{t-1})^2/2)$, and a convolution of two Gaussian functions $\sum_{\theta_{t-1}} \exp(-\sigma(\theta_t - \theta_{t-1})^2/2) \exp\left(-\tilde{\rho}_{t-1}\left(\theta_{t-1} - \hat{\theta}_{t-1}\right)^2/2\right) \propto \exp\left(-\frac{\sigma\tilde{\rho}_{t-1}}{\tilde{\rho}_{t-1}+\sigma}\left(\theta_t - \hat{\theta}_{t-1}\right)^2/2\right)$.) Comparing it to Eq. (S11), we have

$$a_t^i = \alpha_{t-1}\rho_{t-1}^i, \tag{S20}$$

where $\alpha_{t-1} = \sigma/(\tilde{\rho}_{t-1} + \sigma)$ can be implemented by the divisive gain-control mechanism in biological systems [8]. This term is further approximated by a constant value if the ratio $\tilde{\rho}_{t-1}/\sigma$ is within a relatively narrow range; i.e., the uncertainty of momentary posterior and the magnitude of stimulus fluctuation are at roughly the same order, which would be reasonable in practical situations.

Together,

$$\rho_t^i = r_t^i + \alpha \rho_{t-1}^i + \beta f^{\mathrm{cat}}\left(\varphi_i - \varphi_{\hat{c}_{t-1}}^{\mathrm{cat}}\right). \tag{S21}$$

This corresponds to Eq. (4) in the **Results** in main text.. In the present simulations, we computed the quantities appearing in the **Results** section—such as the stimulus discrimination threshold (via the ideal observer analysis, **Methods**) and the mean peak of population activity—based on the spike statistics (mean, variance etc.) determined by the Poisson process, where each neuron's mean spike count is ρ_t^i. Note that, in the current problem setup, the expected value of $\mathbf{r}$ is the sufficient statistic that describes Poisson distribution over spike count variability of each neuron. In our simulation, we set the weights $(\alpha, \beta) = (0.5, 0.2)$ to roughly fit the data for single neuron response modulation (**Fig. 2**); moderate changes in these and other variables (including the width of color selectivity and anisotropy of top-down connectivity) did not affect the main findings of the present paper.

Finally, we describe how the category is estimated based on the neural population activity. The categorical estimate is given by maximizing the posterior based on the history of sensory input:

$$\hat{c}_t = \underset{c_t}{\operatorname{argmax}}\, \mathrm{P}[c_t | D_t] = \underset{c_t}{\operatorname{argmax}} \sum_{\theta_t} \mathrm{P}[c_t | \theta_t, D_t]\, \mathrm{P}[\theta_t | D_t], \tag{S22}$$

which is implemented as a winner-take-all competition in the neural network model (**Methods**). From the assumption that $\mathrm{P}[c_t | \theta_t, D_t]$ is approximated by a von Mises distribution over θ_t that is maximized around $\theta_t = \varphi_{c_t}^{\mathrm{cat}}$, the maximization procedure is well approximated by:

$$\hat{c}_t = \underset{c_t}{\operatorname{argmax}}\, \ln \mathrm{P}\left[\theta_t = \varphi_{c_t}^{\mathrm{cat}} \middle| D_t\right] = \underset{c_t}{\operatorname{argmax}} \sum_i \rho_t^i \ln f\left(\varphi_i - \varphi_{c_t}^{\mathrm{cat}}\right). \tag{S23}$$

Note that this approximation works not only for a von Mises distribution but for a general bell-shaped function $\mathrm{P}[\theta_t | c_t] =$

$g(\theta_t - \varphi_{c_t}^{\mathrm{cat}})$ that peaks at the focal stimulus $\varphi_{c_t}^{\mathrm{cat}}$, which provides a reasonable description of the categorical generative model for color or other stimulus features in general (e.g., orientation, motion, or facial expression). Equation (S23) demonstrates that the category estimate is simply provided by reading out the population activity of hue-selective neurons (ρ_t^i) on each time step, as we implemented in the neural network mode (**Fig. 1d**), This is the case because the instantaneous population activity of hue-selective neurons reflects the history of previous category estimates: as we have described, the population activity of hue-selective neurons is modulated by the previous estimate of category as well as the previous activities of themselves, to represent the posterior distribution of hue ($\mathrm{P}[\theta_t | D_t]$) based on the history of sensory input (D_t) up to that moment.

Table S1. Summary of model parameters used in the simulation.

Description	Notation	Value	Comment
Sharpness of tuning in hue-selective neurons	κ	3	
Sharpness of top-down connectivity from category-selective neurons to hue-selective neurons	κ^{cat}	3	
Strength of lateral interaction (relative to sensory input)	α	0.5	
Strength of top-down interaction (relative to sensory input)	β	0.2	Set β=0 to simulate 'Without top-down' condition in Fig. 2.
Number of hue-selective neurons	N	300	
Number of category-selective neurons	n	3	
Preferred hue of hue-selective neuron i ($i \in \{1, \ldots, N\}$)	φ_i	$-\pi + 2\pi i/N$	Uniformly distributed in angle from $-\pi$ to π (radians).
Preferred hue of category-selective neuron i ($i \in \{1, \ldots, n\}$)	φ_i^{cat}	$-\pi + 2\pi i/n$	

B. Electrophysiological recording and data analysis

Details of the surgical and recording procedures have been previously published [9]. Two female monkeys (*Macaca fuscata*) were used for the experiments. The monkeys were trained in a categorization task, a discrimination task, and a simple fixation task. In all three tasks, 11 sample colors were presented in a pseudorandom order. The monkey was required to maintain fixation within the trial, except for the saccade response. The sample color stimulus was presented for 500 ms. There were eleven sample colors that ranged from red [color 1, (x, y) = (0.631, 0.343)] to green [color 11, (x, y) = (0.286, 0.603)] with equal spaces on the International Commission on Illumination (CIE) xy chromaticity diagram.

In the categorization task, the monkey reported whether the sample color was reddish (sample colors 1–4) or greenish (sample colors 8–11) by saccade, and was rewarded for correct responses. For the intermediate colors (sample colors 5–7), the monkey was rewarded randomly regardless of its behavioral response. In the discrimination task, the monkey reported which test color was the same as the reference color by saccade. The two choice colors were three steps apart along the 11 sample colors: the eight choice-color pairs included colors 1–4, 2–5, 3–6, 4–7, 5–8, 6–9, 7–10, and 8–11. This color interval was chosen so as to yield a modest performance (about 80–90% correct).

Neuronal activity was recorded from the anterior part of the IT cortex, which is a region where color-selective neurons are concentrated. To record single unit activities, microelectrodes were inserted, and the activities of single neurons were isolated by matching spike templates. We analyzed only data from correct trials. The visual response to a sample stimulus was computed as the firing rate between 50 and 550 ms after the sample onset. To determine the neuronal color preferences, we first averaged the firing rates during the above time range, and selected the stimulus color (either of the 11 sample stimulus) that evoked the maximum firing rate for each cell, as its preferred color. In the derivation of the population response distribution, the individual cell responses within each time bin were divided by the time-averaged response to its preferred color during the fixation task. Then, the responses for cells that had the same color preference were respectively averaged. The peak loci of the population responses were obtained by fitting Gaussian functions with variable mean, variance, gain, and baseline level. The responses to the marginal colors (1 and 11 for the categorization task; 1, 2, and 11 for the fixation task) were excluded from the peak analysis because the peak estimates were not reliable for these stimuli. To test the statistical significance of the population activity modulation, we conducted 3-way analysis of variance (ANOVA; preferred stimulus$\times$presented stimulus$\times$time) on the difference

between the activities during categorization and discrimination tasks.

All procedures for animal care and experimentation were in accordance with the National Institutes of Health Guide for the Care and Use of Laboratory Animals and were approved by Institutional Animal Care and Use Committee of the National Institute of Natural Sciences.

References

1. Yu, A. J. & Dayan, P. Acetylcholine in cortical inference. *Neural Networks* **15,** 719–30 (2002).

2. Goris, R. L. T., Movshon, J. A. & Simoncelli, E. P. Partitioning neuronal variability. *Nat. Neurosci.* **17,** 858–865 (2014).

3. Földiák, P. in *Computation and Neural Systems* (eds. Eeckman, F. H. & Bower, J. M.) **1992,** 55–60 (Norwell, MA: Kluwer Academic Publishers, 1993).

4. Sanger, T. Probability density estimation for the interpretation of neural population codes. *J. Neurophysiol.* **76,** 2799–2793 (1996).

5. Dayan, P. & Abbott, L. F. *Theoretical Neuroscience: Computational and Mathematical Modeling of Neural Systems*. (MIT Press., 2001).

6. Ma, W. J., Beck, J. M., Latham, P. E. & Pouget, A. Bayesian inference with probabilistic population codes. *Nat. Neurosci.* **9,** 1432–1438 (2006).

7. Jazayeri, M. & Movshon, J. A. Optimal representation of sensory information by neural populations. *Nat. Neurosci.* **9,** 690–696 (2006).

8. Beck, J. M., Latham, P. E. & Pouget, A. Marginalization in neural circuits with divisive normalization. *J. Neurosci.* **31,** 15310–15319 (2011).

9. Koida, K. & Komatsu, H. Effects of task demands on the responses of color-selective neurons in the inferior temporal cortex. *Nat. Neurosci.* **10,** 108–16 (2007).

Supplementary Figures

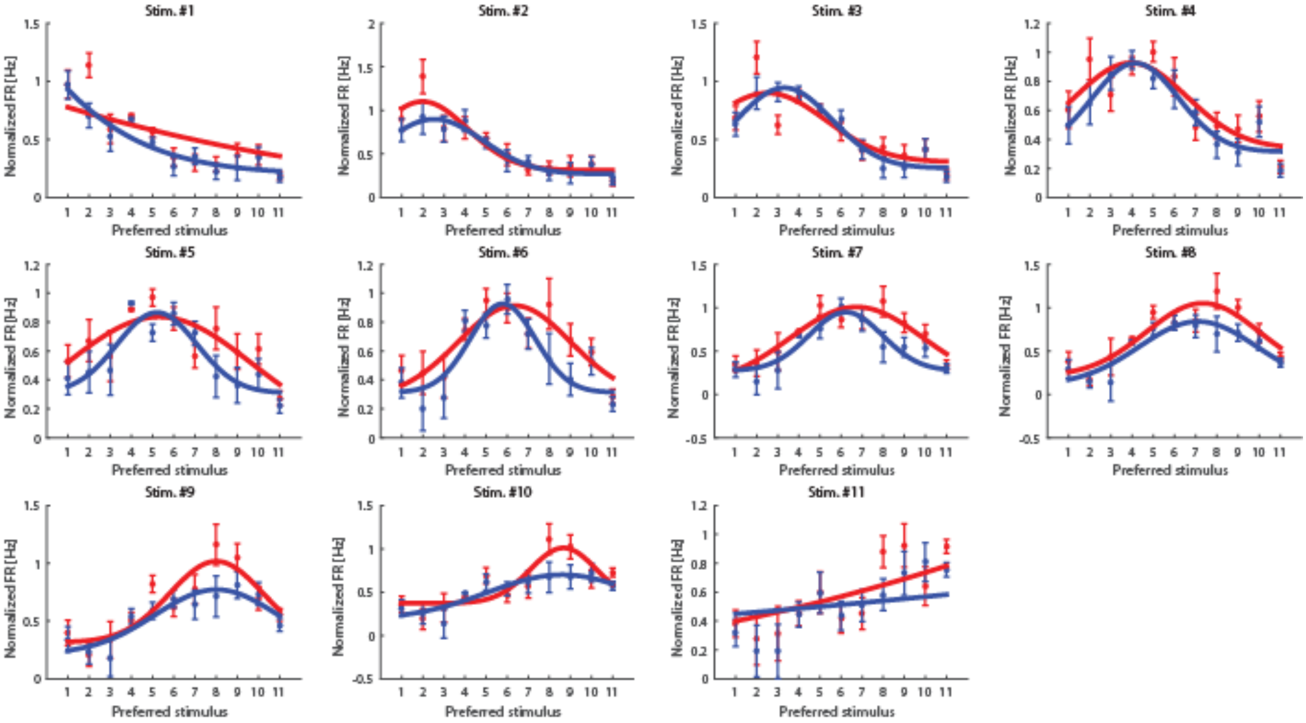

Supplementary Figure S1.

Modulation of the population activity during the categorization and discrimination tasks for all visual stimuli (color 1–11). The conventions follow **Fig. 5a**.

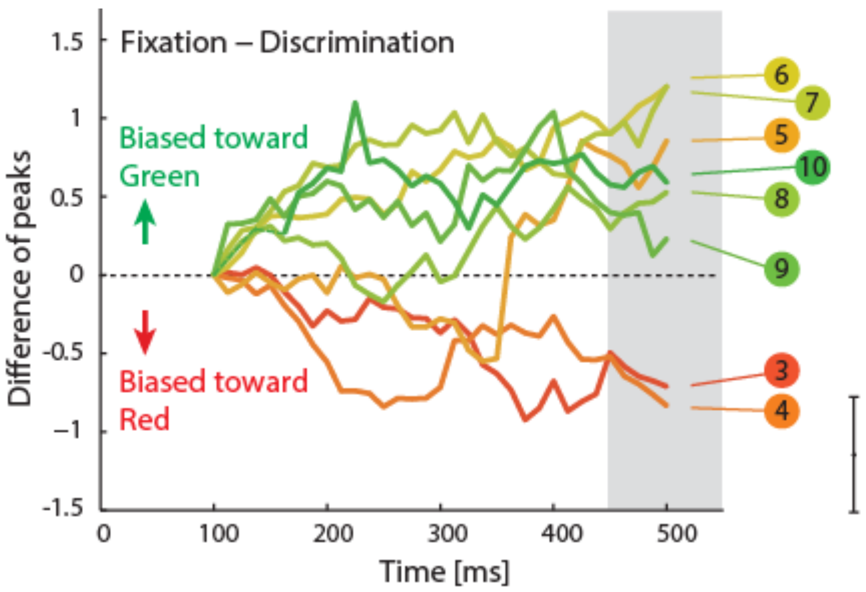

Supplementary Figure S2.

The temporal evolution of population peak difference between fixation and discrimination tasks. The presentation conventions follow **Fig. 5c**.

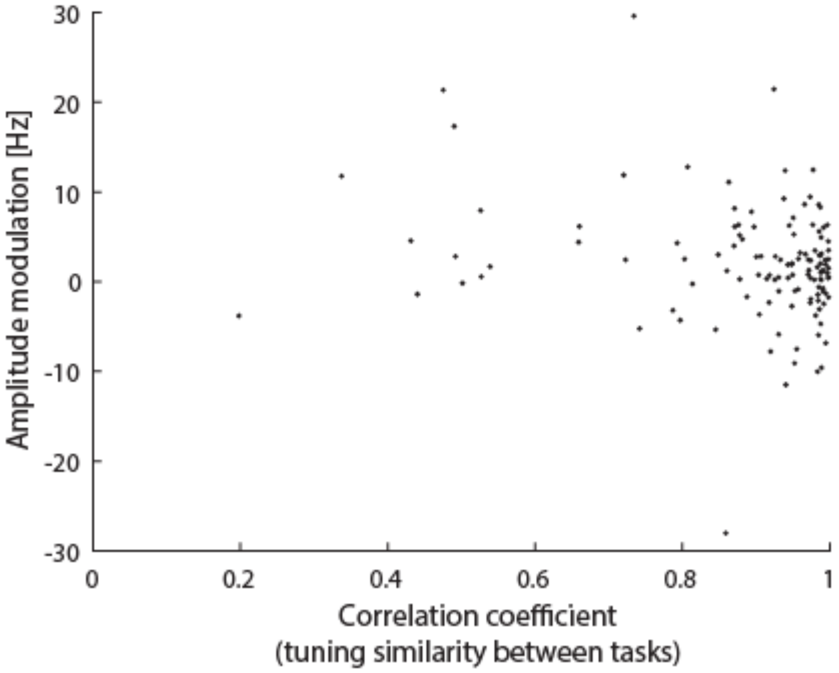

Supplementary Figure S3.

The response amplitude modulation (categorization – discriminaiton) is compared with the tuning curve correlation between the tasks (categorization vs. discrimination). Each dot indicates a single neuron. The correlation coefficient (the horizontal axis) and the amplitude modulation (the vertical axis) are the same as the ones used in **Figs. 2b** and **2c**, respectively.
